# Acute and Prolonged Effects of Dermal Suction on Joint Range of Motion and Passive Muscle Stiffness: A Preliminary Study

**DOI:** 10.3390/healthcare10112241

**Published:** 2022-11-09

**Authors:** Shota Enomoto, Tomonari Shibutani, Yu Akihara, Kazunori Yamada, Toshiaki Oda

**Affiliations:** 1 Institute for Promotion of Education and Campus Life, Okayama University, 2-1-1 Tsusima-naka, Kita-ku, Okayama 700-8530, Japan; 2 Center for Liberal Arts, Meiji Gakuin University, 1518, Kamikurata-cho, Totsuka-ku, Yokohama 244-8539, Japan; 3 Institute of Sports Sciences, International Pacific University, 721 Kannonji, Seto-cho, Higashi-ku, Okayama 709-0863, Japan; 4 MJ Company K.K., 308-6, Nishiichi, Minami-ku, Okayama 700-0953, Japan; 5 Fourleaf K.K., 308-6, Nishiichi, Minami-ku, Okayama 700-0953, Japan; 6 Graduate School of Education, Hyogo University of Teacher Education, 942-1, Shimokume Kato, Hyogo 673-1494, Japan; 7 Faculty of Health and Well-Being, Kansai University, Sakai 590-8515, Japan; 8 Department of Community Child Studies, Niijima Gakuen Junior College, 53, Showa-machi, Gunma, Takasaki-city 370-0068, Japan; 9 Department of Education, Tokai Gakuen University, Nagoya 468-8514, Japan

**Keywords:** elastography, flexibility, rectus femoris, shear wave velocity, range of motion, ultrasound, vastus lateralis

## Abstract

This study aimed to investigate the acute and prolonged effects of dermal suction on joint range of motion (ROM) and passive muscle stiffness. Eight-minute dermal suction was prescribed for the quadriceps femoris in 15 participants. Hip extension ROM, knee flexion ROM, and passive muscle stiffness of the rectus femoris (RF) and vastus lateralis (VL) were measured before and immediately, 30 min, 60 min, 120 min, 24 h, and 48 h after dermal suction. Passive muscle stiffness was measured using shear wave elastography. Hip extension ROM significantly increased immediately (*p* = 0.032), 60 min (*p* = 0.029), and 120 min (*p* = 0.031) after dermal suction compared with before dermal suction; however, it was not significantly different at 30 min, 24 h, and 48 h after dermal suction (*p* > 0.05). Passive muscle stiffness of the RF and VL and knee flexion ROM did not significantly change at any measurement time compared with before dermal suction (*p* > 0.05). Our preliminary results suggest that dermal suction improves hip extension ROM immediately after dermal suction of the quadriceps femoris, followed by a return to the pre-prescription level 30 min after. However, the effect was prolonged for 120 min and disappeared before 24 h.

## 1. Introduction

Flexibility, a component of physical fitness, is believed to be an important factor in sports performance and injury prevention. Dermal suction is one of several methods for improving flexibility. Dermal suction treatment is called cupping therapy in traditional Chinese medicine. In cupping therapy, a vacuum is applied to a targeted skin area [1]. Several researchers have investigated changes in flexibility due to cupping therapy; furthermore, some found significant increases in the joint range of motion (ROM) after cupping therapy [2,3,4,5,6], while others discovered that cupping therapy did not significantly change the joint ROM [7,8].

In the aforementioned studies, joint ROM was used as an index of joint flexibility. However, all structures around the joint, such as the skin, muscles, and tendons, contribute to joint flexibility [9]. Therefore, in previous research targeting only joint flexibility, what caused the change in joint ROM due to dermal suction was not clear. Focusing on this point, Enomoto et al. (2021) attempted to elucidate part of the mechanism of joint flexibility improvement by dermal suction [10]. Enomoto et al. (2021) measured the stiffness of the medial gastrocnemius (MG) immediately before and after dermal suction of the calf using shear wave elastography and discovered that passive muscle stiffness significantly increased immediately after dermal suction [10]. However, in their study [10], the measurement time was limited to immediately after dermal suction.

The prolonged effects of dermal suction intervention are important for strength and conditioning coaches or physical therapists who perform dermal suction in clinical practice on joints or muscles. Stretching [11,12] and foam rolling [13] are used to target joints and muscles in a manner similar to dermal suction, and have been studied for their acute and prolonged effects. Research has examined the prolonged changes in joint ROM by cupping therapy [7,8]; however, muscle stiffness has not been assessed. Examining the prolonged effects of dermal suction on joint ROM and muscle stiffness can elucidate some of the mechanisms underlying the effects of dermal suction on joint ROM and provide beneficial knowledge for strength and conditioning coaches or physical therapists. Therefore, the purpose of this study was to examine the acute and prolonged effects of dermal suction on joint ROM and muscle stiffness. We hypothesized that joint ROM and muscle stiffness increase acutely and long-term after dermal suction.

## 2. Materials and Methods

### 2.1. Participants

We recruited participants among students and researchers at the investigator’s university and received details of the experiment at the time of recruitment. A total of 30 people received the information, of which 15 gave their consent to participate in this study. Participants comprised seven men and eight women who were recreationally active. They did not participate in competitive athletic events, and regular resistance training did not exceed 3 times per week. We requested the participants refrain from strenuous exercise from 24 h before the examination. No participants complained of any injuries specific to the leg. We explained the purpose, content, methods, and risks of the study orally and in writing before the examination. We obtained written informed consent from all the participants. The local ethics committee approved this study (approval number: 2019-2).

### 2.2. Procedure

We designed this single-arm study to investigate the acute and prolonged effects of dermal suction on joint ROM and passive stiffness of muscle. In this study, we targeted the right leg in all participants. We measured joint ROM of the hip and knee and passive muscle stiffness of the rectus femoris (RF) and vastus lateralis (VL) before (PRE) and immediately (POST), 30 min, 60 min, 120 min, 24 h, and 48 h after 8 min of dermal suction, which we targeted to the right quadriceps femoris.

### 2.3. Joint ROM

**Figure 1:**
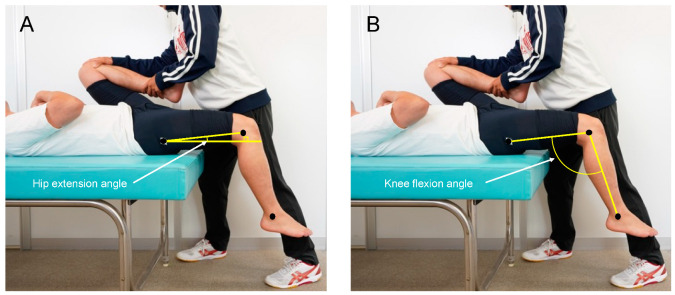
Measurement and calculation of (**A**) hip extension and (**B**) knee flexion joint angles.

We measured hip extension and knee flexion ROMs as indices of joint flexibility. We measured these ROMs using the modified Thomas test [14]. The modified Thomas test has been used in previous studies to examine changes in hip extension ROM or knee flexion ROM when foam rolling is prescribed for the front thigh [15,16]. The participants sat at the end of the massage table, rolled back onto the table, and held both knees to their chest [14]. The investigator held the left hip in maximal flexion while lowering the right limb towards the floor (Figure 1). The investigator who performed hip holding in the Thomas test was a researcher with more than 10 years of experimental experience in the biomechanics field.

We photographed all tests from the sagittal plane (EXILIM EX-100, CASIO Inc., Tokyo, Japan) and analyzed using open source software (Kinovea, version 0.8.27). We marked three anatomical landmarks (the greater trochanter, lateral femoral epicondyle, and lateral malleolus) to obtain the hip and knee angles. We defined hip extension ROM as the angle formed between a line of the lateral femoral epicondyle and greater trochanter and a horizontal reference line. In addition, we defined knee flexion ROM as the angle formed between a line with respect to the greater trochanter and the lateral femoral epicondyle and a line with respect to the lateral malleolus and lateral femoral epicondyle. We performed each ROM measurement twice and utilized the average of both values for analysis.

### 2.4. Passive Muscle Stiffness

After measuring the joint ROM, we measured the passive muscle stiffness of the RF and VL. During the measurements, the participants lay in a supine position with their knees fully extended and were instructed to relax all their muscles. We used an ultrasound shear wave elastography scanner (Aixplorer; Supersonic Imagine, Aix-en-Provence, France) with a linear array transducer (SL15-4; Supersonic Imagine, France) to measure the passive stiffness of muscle. We used shear wave velocity (SWV) (m/s) as an index of muscle stiffness. We placed the probe at the midpoint of the femur (from the greater trochanter to the lateral epicondyle of the femur) for RF and VL. We measured the SWV twice for each muscle. The measurements of the SWV were performed by a researcher with more than 10 years of experimental experience with ultrasonography. We calculated the SWV over the region of interest, which we manually set for RF and VL. We used ultrasound shear wave elastography scanner built-in software (Q-box) to analyze SWV. We averaged the values of three images and utilized the average value of each measurement for the analysis.

### 2.5. Dermal Suction

**Figure 2:**
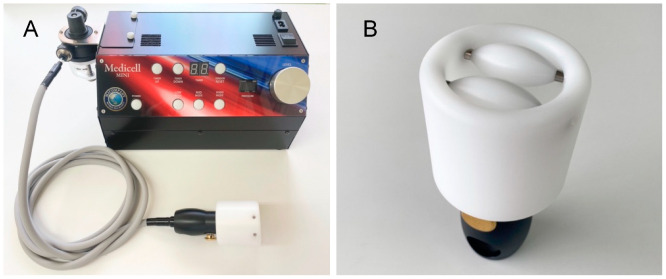
The equipment (Medicell) used to perform dermal suction. The device comprises (**A**) a part that generates negative pressure and (**B**) a cup that provides suction to the skin. The cup has a roller to improve cup sliding.

In dermal suction, we referred to Enomoto et al. (2021), who examined the acute effects of dermal suction on passive joint and muscle stiffness [10]. We used Medicell (MJ Company K.K., Okayama, Japan) to perform dermal suction (Figure 2). This device has been used in human and animal research for dermal suction [10,17]. This device consists of a cup that provides suction to the skin and a part that generates negative pressure. We performed a total of 8 min of dermal suction on the right quadriceps femoris (4 min, followed by a 30 s rest and 4 min more). During the treatment, we instructed the participants to relax their whole bodies while lying supine on a massage table. We slid the cup from 10–15 cm distal to the iliac crest to approximately 5 cm proximal to the patella at approximately 0.5 Hz during the dermal suction. We set the intensity to 20 kPa of negative pressure and corrected before each treatment session. To improve cup sliding, we applied baby oil (Johnson & Johnson K.K., Tokyo, Japan) consisting of tocopherol acetate and mineral oil to the targeted area.

### 2.6. Reliability

Prior to the examination, we conducted a pilot study using five participants to assess reliability. We twice measured hip extension and knee flexion ROM and passive muscle stiffness of the RF and VL for each participant, and then we calculated intraclass correlation coefficients (ICC) and 95% confidence intervals (CIs).

### 2.7. Statistics

We performed linear mixed models using RStudio statistical software version 1.4.1106 (R Core Team 2021, Vienna, Austria) [18]. We added sex and participants as random effects to the models. First, we implemented models that included the fixed effect of interest (time) and random effects. Thereafter, we simplified the models by removing non-significant factors (*α* > 0.05) from the least-significant ones with a backward stepwise deletion procedure to arrive at a minimum adequate model [19]. We evaluated the statistical significance of the fixed effect using a likelihood ratio test to compare the change in deviance between the models [20,21]. To verify whether dermal suction changes joint ROM and SWV, we tested the difference between PRE and the other conditions. In multiple comparisons, we used Bonferroni correction to adjust significant thresholds. We set the level of significance at *p* < 0.05.

## 3. Results

The age and physical characteristics of the participants are presented in Table 1. The ICCs for hip extension ROM, knee flexion ROM, passive muscle stiffness of RF, and passive muscle stiffness of VL were 0.94 (95% CIs [0.55, 0.99]), 0.93 (95% CIs [0.51, 0.99]), 0.89 (95% CIs [0.22, 0.99]), and 0.96 (95% CIs [0.71, 1.00]), respectively, and were considered “almost perfect” [22].

**Figure 3:**
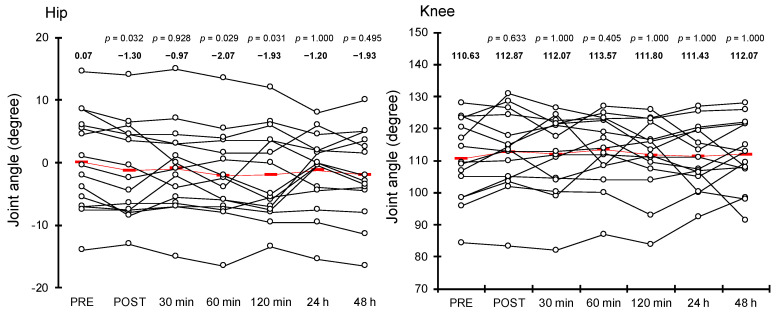
Mean (red rectangle) and individual (white circle) values of hip extension and knee flexion range of motion at each measurement time. We showed the *p* value when compared with “PRE” at each measurement time at the top of the graph. We also showed the mean value of each measurement above each plot.

The hip extension ROM for each measurement is shown in Figure 3. We found a significant increase in hip extension ROM at POST (χ*^2^* = 7.777, *df* = 1, *p* = 0.032), 60 min (χ*^2^* = 7.915, *df* = 1, *p* = 0.029), and 120 min (χ*^2^* = 7.833, *df* = 1, *p* = 0.031) after treatment compared with PRE. However, hip extension ROM at 30 min (χ*^2^* = 2.026, *df* = 1, *p* = 0.928), 24 h (χ*^2^* = 1.639, *df* = 1, *p* = 1.000), and 48 h (χ*^2^* = 3.015, *df* = 1, *p* = 0.495) after treatment revealed no significant differences compared with PRE.

The knee flexion ROM for each measurement is depicted in Figure 3. There were no significant changes in any post-treatment values of knee flexion ROM compared with PRE (POST: χ*^2^* = 2.621, *df* = 1, *p* = 0.633; 30 min after treatment: χ*^2^* = 0.638, *df* = 1, *p* = 1.000; 60 min after treatment: χ*^2^* = 3.343, *df* = 1, *p* = 0.405; 120 min after treatment: χ*^2^* = 0.558, *df* = 1, *p* = 1.000; 24 h after treatment: χ*^2^* = 0.220, *df* = 1, *p* = 1.000; and 48 h after treatment: χ*^2^* = 0.297, *df* = 1, *p* = 1.000).

**Figure 4:**
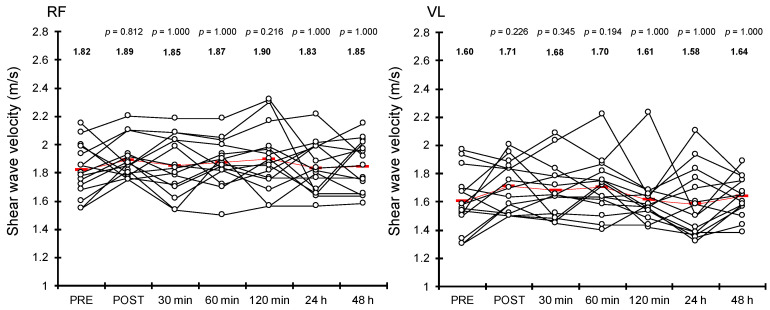
Mean (red rectangle) and individual (white circle) values of shear wave velocity of rectus femoris (RF) and vastus lateralis (VL) at each measurement time. We showed the *p* value when compared with “PRE” at each measurement time at the top of the graph. We also showed the mean value of each measurement above each plot.

Figure 4 shows the SWV of RF for each measurement. All post-treatment values of passive muscle stiffness of RF demonstrated no significant changes compared with PRE (POST: χ*^2^* = 2.230, *df* = 1, *p* = 0.812; 30 min after treatment: χ*^2^* = 0.919, *df* = 1, *p* = 1.000; 60 min after treatment: χ*^2^* = 1.816, *df* = 1, *p* = 1.000; 120 min after treatment: χ*^2^* = 4.398, *df* = 1, *p* = 0.216; 24 h after treatment: χ*^2^* = 0.093, *df* = 1, *p* = 1.000; and 48 h after treatment: χ*^2^* = 0.360, *df* = 1, *p* = 1.000).

The SWV of the VL for each treatment is shown in Figure 4. We found no significant change in any post-treatment value of SWV of VL compared with PRE (POST: χ*^2^* = 4.319, *df* = 1, *p* = 0.226; 30 min after treatment: χ*^2^* = 3.609, *df* = 1, *p* = 0.345; 60 min after treatment: χ*^2^* = 4.581, *df* = 1, *p* = 0.194; 120 min after treatment: χ*^2^* = 0.103, *df* = 1, *p* = 1.000; 24 h after treatment: χ*^2^* = 0.082, *df* = 1, *p* = 1.000; and 48 h after treatment: χ*^2^* = 0.589, *df* = 1, *p* = 1.000).

## 4. Discussion

In this study, we compared joint ROM and passive muscle stiffness before and immediately, 30 min, 60 min, 120 min, 24 h, and 48 h after 8 min of dermal suction and obtained the following main findings. Dermal suction increased hip extension ROM immediately, 60 min, and 120 min after dermal suction compared with baseline; however, it was not significantly different at 30 min, 24 h, and 48 h after dermal suction. On the other hand, passive muscle stiffness of both the RF and VL and knee flexion ROM did not change significantly at any measurement time compared with the pre-treatment level. 

The results revealed that hip extension ROM increased significantly immediately, 60 min, and 120 min after dermal suction but not at 30 min, 24 h, and 48 h after dermal suction (Figure 3). Some possible explanations exist for the acute and prolonged increase in hip extension ROM after dermal suction. A previous study reported a significant decrease in skin stiffness following cupping therapy [23]. The skin is one of the factors that are related to joint ROM [24]; hence, a reduction in skin stiffness caused by dermal suction may have contributed to the improvement of joint ROM in this study. Muscle mechanical properties are also associated with joint flexibility [25]. A decrease in stiffness of the RF could have contributed to the increase in hip extension ROM observed in this study because RF contributes to hip extension ROM. However, the SWV of RF showed non-significant changes in this study (Figure 4). This suggests that the decreases in skin stiffness described above, or the changes in other tissues, such as the tendons, and other muscles affecting hip extension ROM, such as the iliopsoas, were related to the observed increases in hip extension ROM. Further studies, including of the muscles and other tissues related to joint ROM, such as tendons and skin, would provide beneficial information on the mechanisms of the acute and prolonged effects of dermal suction on joint ROM.

As mentioned above, hip extension ROM increased significantly immediately, 60 min, and 120 min after dermal suction, with changes ranging from 1.37° to 2.14° when compared with those before dermal suction (Figure 3). Chiacchiero et al. (2010) investigated the relationship between joint ROM and falls and reported that hip extension ROM in elderly participants with two or more falls over the past 12 months was 3.56° lower compared with that of elderly without falls over the past 12 months [26]. Another study prescribed 10 weeks of stretching exercise for the hip flexor muscle group in elderly participants and reported that hip extension ROM improved significantly by 1.6° after intervention [27]. These reports taken together suggest that an increased hip extension ROM of 1.37° to 2.14° can be considered a meaningful effect. On the other hand, it should be noted that the participants of the aforementioned studies were elderly, while those in this study were young adults.

In contrast to hip extension ROM, knee flexion ROM showed no significant changes at any measurement time after dermal suction compared with that before dermal suction (Figure 3). It is difficult to explain the mechanism of this phenomenon; however, one possibility is that there are differences in factors related to hip and knee joint flexibility. Joint flexibility is contributed to by all structures around the joint, such as the skin, muscles, and tendons [9]. Although speculative, the factors strongly related to hip extension ROM and knee flexion ROM differ. Dermal suction may have a greater effect on the factors that are strongly related to hip extension ROM, which could explain the different results for the two joints in this study.

In our study, passive muscle stiffness of both the RF and VL showed no significant changes at any measurement time after dermal suction compared with that before dermal suction (Figure 4). These results are inconsistent with those of previous research that observed a significant increase in passive muscle stiffness immediately after dermal suction [10]. This discrepancy may be due to differences in the target muscle groups. In this study, dermal suction was prescribed for the quadriceps femoris, and we measured the passive stiffness of the RF and VL. Enomoto et al. prescribed dermal suction for the calf and measured the MG passive stiffness [10]. The quadriceps femoris and triceps surae differ in the number of muscles they compose and in their total volume (quadriceps femoris, 1791 cm^3^; triceps surae, 819 cm^3^) [28]. In addition, previous studies have reported that the subcutaneous adipose tissue of the front thigh is approximately 1.6 times thicker than that of the medial calf (see Table 4 in Ref. [29]). These characteristics of the targeted muscle groups and surrounding tissues could have influenced the changes in passive muscle stiffness after dermal suction. The position at which passive muscle stiffness was measured might also be related to the discrepancy between this study and a previous study [10]. This study measured the passive muscle stiffness of the RF and VL in the fully extended knee position. In this position, the targeted muscles were not stretched and were considered slack [30]. On the other hand, Enomoto et al. measured the passive muscle stiffness of the MG immediately before and after dermal suction in four positions: 20°, 10°, 0°, and −10°, where 0° represented the neutral ankle position, positive values represented plantar flexion angles, and negative values represented dorsiflexion angles [10]. According to previous studies that measured the slack angle of the triceps surae, the slack angle of the MG has been reported to be approximately 20° plantar flexion (Ref. [31]: 20.7° (SD 6.7); Ref. [32] 19.1° (SD 4.9); Ref. [33] 24.3° (SD 5.8)). Thus, in the position used by Enomoto et al., the targeted muscles could exceed the slack angle [10]; hence, the muscles could be stretched. Therefore, the condition of the muscle (stretched or unstretched) attributed to the position at the time of measurement may have resulted in the different results in this study and the previous study [10].

This study had some limitations. First, this study was a single-arm study with no control group, making it difficult to determine whether the improvements in hip extension ROM were due to the dermal suction treatment, the effects of natural history, or a placebo effect [34]. Future studies with control groups are needed to accurately assess the effects of dermal suction on joint ROM or passive muscle stiffness. Second, the duration and intensity of dermal suction were limited. Experiments with various intensities, durations, or combinations of these factors would provide useful data on the effects of dermal suction on joint ROM and passive muscle stiffness.

## 5. Conclusions

This preliminary study provides information regarding acute and prolonged changes in passive muscle stiffness and joint ROM after dermal suction. Our results suggest that hip extension ROM improves immediately after dermal suction of the quadriceps femoris, followed by a return to baseline 30 min later. However, the effect was prolonged for 120 min and disappeared before 24 h. Moreover, our results also suggest that dermal suction has limited acute and prolonged effects on knee flexion ROM and passive muscle stiffness of the RF and VL. These results provide preliminary findings to design better future studies on dermal suction.

## Figures and Tables

**Table 1:** Age and physical characteristics of the participants.

Sex	Age (Years)	Height (cm)	Body Mass (kg)
Men	28.0 (4.5)	172.1 (3.1)	72.2 (15.0)
Women	22.8 (1.5)	158.2 (4.5)	54.9 (8.3)

## Data Availability

The data for this study are available from the corresponding author upon reasonable request.
